# Transcriptome analysis identifies LGP2 as an MDA5-mediated signaling activator following spring viremia of carp virus infection in common carp (*Cyprinus carpio* L.)

**DOI:** 10.3389/fimmu.2022.1019872

**Published:** 2022-10-18

**Authors:** Rongrong Liu, Yan Niu, Yue Qi, Hua Li, Guiwen Yang, Shijuan Shan

**Affiliations:** Shandong Provincial Key Laboratory of Animal Resistance Biology, College of Life Sciences, Shandong Normal University, Jinan, China

**Keywords:** *Cyprinus carpio* L., spring viremia of carp virus (SVCV), transcriptome analysis, MDA5, LGP2

## Abstract

The common carp (*Cyprinus carpio* L.) is an important farmed species worldwide. Mucosal-associated lymphoid tissues play an essential role in the fight against pathogen infection. Spring viremia of carp virus (SVCV) poses a serious threat to the common carp aquaculture industry. Understanding the molecular mechanisms driving mucosal immune responses to SVCV infection is critical. In this study, the mucosal tissues (gills, foregut and hindgut) were collected from normal and infected fishes for transcriptome analysis. A total of 932,378,600 clean reads were obtained, of which approximately 80% were successfully mapped to the common carp genome. 577, 1,054 and 1,014 differential expressed genes (DEGs) were identified in the gills, foregut and hindgut, respectively. A quantitative polymerase chain reaction assay indicated that the DEGs expression in the foregut following SVCV infection was consistent with the transcriptome results. Among them, two key genes of the retinoic acid-inducible gene I (RIG-I)-like receptor family, melanoma-differentiation-associated gene 5 (MDA5) and laboratory of genetics and physiology 2 (LGP2) (i.e., *Cc*MDA5 and *Cc*LGP2), underwent further analysis. Overexpression of *Cc*MDA5 or *Cc*LGP2 increased phosphorylation of TANK-binding kinase 1 and interferon regulatory factor 3 and the expression of *interferon-1* (*ifn-1*), *myxovirus resistance* (*mx*), *viperin* and *interferon-stimulated gene 15* (*isg15*), and inhibited SVCV replication in epithelioma papulosum cyprini cells. Furthermore, *Cc*LGP2 significantly upregulated the *Cc*MDA5-induced *ifn-1* mRNA expression and the activation of the *ifn-1* promoter. Finally, confocal microscopy and coimmunoprecipitation experiments revealed that *Cc*LGP2 colocalizes and interacts with *Cc*MDA5 *via* the C-terminal regulatory domain. This study provides essential gene resources for understanding the fish immune response to SVCV infection and sheds light on the potential role of fish LGP2 in the MDA5 regulation.

## Introduction

Spring viremia of carp virus (SVCV) belongs to the *Vesiculovirus* genus of the *Rhabdoviridae* family (1). It is a linear, negative-sense, single-stranded RNA virus that is believed to be the cause of spring viremia of carp (2). Affected fish suffer from acute hemorrhagic illness and have a high mortality rate (3). Once infected, it is difficult to eradicate the virus from affected ponds (4). Currently, no specific medicine is approved for the treatment of SVCV-induced diseases, nevertheless, safe and effective vaccines are being developed. Hence, it is critical to explore the intricate mechanisms of host-virus interaction.

The innate immune cells utilize pattern recognition receptors (PRRs) to sense invading viruses (5) (6). The retinoic acid-inducible gene-I (RIG-I)-like receptors (RLRs) are the key intracellular PRRs that can detect viral RNA (7), including RIG-I, melanoma differentiation-associated protein 5 (MDA5) and laboratory of genetics and physiology 2 (LGP2) (8). Upon RNA virus recognition, RIG-I and MDA5 undergo conformational changes and then recruit and interact with a mitochondrial adaptor protein (MAVS) (9). MAVS aggregation transmits a signal to downstream signaling molecules, activating the TANK-binding kinase 1 (TBK1) and the inhibitor of nuclear factor kappa-B kinase epsilon (IKKϵ). These kinases, in turn, activate interferon regulatory factor 3 (IRF3) and nuclear factor-kappa B (NF-κB), leading to the production of interferon (IFN) and proinflammatory cytokine (10).

LGP2, also called DExH-Box helicase 58 (DHX58), is the third member of RLRs (11). Owing to the lack of N-terminal caspase activation and recruitment domains (CARD), it is widely believed to regulate RIG-I and MDA5 *via* different mechanisms (12–14). LGP2 was initially identified as a negative regulator of RIG-I and MDA5 signaling. For instance, LGP2 interacts with RIG-I *via* its C-terminal regulatory domain (RD) to block RIG-I self-association and signaling (15). In contrast, LGP2-deficient mice have a positive regulatory role in RIG-I- and MDA5-mediated antiviral responses (16). So far, RLR orthologs have been cloned and characterized from various fish species (17). However, the molecular mechanism of LGP2 regulation in RIG-I and MDA5 remains inconsistent in teleost. For instance, black carp LGP2 associates with RIG-I/MDA5 to activate IFN signaling and the host antiviral innate response (18). Nonetheless, grass carp LGP2 inhibits IFN-β promoter stimulator 1 and IRF3 activation *via* RIG-I and MDA5 (19). Hence, it is important to determine the role of fish LGP2 in RIG-I/MDA5-mediated antiviral response.

Common carp (*Cyprinus carpio* L.), an important freshwater aquaculture species worldwide, is facing disease outbreak problems from bacterial, parasitic, and viral pathogens (20, 21). Particularly, SVCV poses a serious threat to common carp health (22, 23). Fish mucosal tissues, such as gills and gut, act as a protective barrier where pathogens against pathogen invasion in the aquatic environment (24). Hence, in this study, RNA-seq technology was utilized to describe differentially expressed genes (DEGs) in the gills, foregut and hindgut of common carp challenged by SVCV. Our results demonstrated that SVCV infection elicited a strong immune response in the mucosal tissues. *Cc*MDA5 and *Cc*LGP2 were selected for further research among these DEGs. Their antiviral activities and regulatory mechanisms were specifically investigated. The study emphasized the fish mucosal immune response to SVCV and will provide new insights into the potential role of fish LGP2 in the MDA5 regulation.

## Material and methods

### Animal samples and viral challenge

Common carp (*C. carpio* L.) with a body weight of 150−200 g were obtained from a fish farm in Jinan and adapted for at least one week before being used in experiments. SVCV were kindly gifted from Yuguang Qin (Shandong Freshwater Fisheries Research Institute, China) and quantified by our laboratory according to previous research (25). The fishes were randomly divided into SVCV-infected and control groups. Carps in the infected group were intraperitoneally injected with 500 μL of SVCV (10^6^ TCID_50_/mL), and the control group received an equal volume of Medium 199 (Gibco, Grand Island, NY, USA). Three fishes were randomly selected from the control and infected groups and immersed in a 100 mg/L-Tricaine Methane Sulfonate solution. Additionally, carp gills, foregut and hindgut samples were collected at 72 h post SVCV or Medium 199 injection. The samples were promptly frozen in liquid nitrogen and stored at -80°C until use.

### RNA isolation, library construction and sequencing

Total RNA was isolated using TRNpure Total RNA Kit (Nobelab, Beijing, China) following the manufacturer’s protocol. The quantity and purity of total RNA were determined using NanoDrop 2000 (Thermo Fisher, Waltham, MA, USA) and Agilent 2100 Bioanalyzer (Agilent Technologies, Palo Alto, CA, USA), respectively. RNA purification, reverse transcription, library construction and sequencing were performed at Shanghai Majorbio Bio-pharm Biotechnology Co., Ltd. (Shanghai, China) according to the manufacturer’s instructions (Illumina, San Diego, CA, USA). The transcriptome library was prepared following the TruSeqTM RNA sample preparation Kit from Illumina (San Diego, CA, USA) using 1 μg of total RNA. The messenger RNA was isolated using oligo(dT) beads and fragmented by fragmentation buffer. The double-stranded complementary DNA (cDNA) was synthesized using a SuperScript double-stranded cDNA synthesis kit (Invitrogen, Carlsbad, CA, USA) with random hexamer primers (Illumina, San Diego, CA, USA) and subjected to end-repair, phosphorylation, and ‘A’ base addition per Illumina’s library construction protocol. Libraries were size selected for cDNA target fragments of 300 bp on 2% Low Range Ultra Agarose followed by PCR amplification including 15 PCR cycles, using Phusion DNA polymerase (NEB, Ipswich, MA, USA). After being quantified by TBS380, the paired-end RNA-seq sequencing library was sequenced with the Illumina NovaSeq 6000 sequencer (2 × 150 bp read length).

### Identification and functional enrichment analysis of DEGs

Raw reads were first quality checked by Sickl (https://github.com/najoshi/sickle) and SeqPrep (https://github.com/jstjohn/SeqPrep) to eliminate adaptor sequence, low-quality sequence and reads containing polyN. The trimmed reads were assessed by fastp v0.19.5, and the high-quality clean reads were aligned with the reference genome of *C. carpio* (GCF_000951615.1) using TopHat Version v2.1.1. The clean data were analyzed on the Majorbio Cloud Platform as previously described (26). Briefly, HISAT2 software was used to map the clean reads to the common carp genome. The mapped reads were assembled using StringTie software. To identify DEGs between the control and SVCV groups (three replicates in each group), TPM (transcripts per million) were used to assess the gene expression. DEGs analysis between two groups was done using DESeq2 software. A false discovery rate (FDR) < 0.05 and |log2 (Fold Change, FC)| ≥1 were used as the threshold for the selection of the DEGs. Lastly, functional-enrichment analysis including Gene Ontology (GO, http://www.geneontology.org) and Kyoto Encyclopedia of Genes and Genomes (KEGG, http://www.genome.jp/kegg/) were performed to identify DEGs that were significantly enriched in GO terms and metabolic pathways at P-adjust ≤ 0.05 when compared to the whole-transcriptome background. GO functional enrichment and KEGG pathway analysis were performed using Goatools (https://github.com/tanghaibao/Goatools) and KOBAS (http://kobas.cbi.pku.edu.cn/home.do).

### Quantitative polymerase chain reaction

Total RNA was extracted from tissues and cells using TRNpure Total RNA Kit (Nobelab, Beijing, China), and 2 μg of total RNA was reverse-transcribed using ReScriptTM II RT SuperMix for qPCR (+gDNA Eraser) (Nobelab, Beijing, China) following the manufacturer’s instructions. Subsequently, the gene expression was detected using 2 × SYBR qPCR Mix (Nobelab, Beijing, China) and performed on a LightCycler 96 instrument (Roche, Switzerland). The qPCR was performed as follows: 95°C for 120 s followed by 40 cycles of 95°C for 10 s and 60°C for 30 s. The relative expression of genes was analyzed using the 2^−ΔΔCT^ method. The primers used in this study were listed in **Table 1**.

**Table 1:** Primers used in this study.

Primer name	Primer (5′→3′)	Application
LGP2-GFP-F	GGGGTACCATGGACTTCAGTCTTAGACC	Plasmid
LGP2-GFP-R	CCCCCGGGTCAGTCGTTTAAGTTTAGGT	Plasmid
MDA5-GFP-F	CCAAGCTTCGATGAGCTGCGATAAGGAC	Plasmid
MDA5-GFP-R	CGGGATCCTCAGTCAGTTTCCAAGTTTT	Plasmid
MDA5-FUGW-F	ACATCCTGCAGGAATGAGCTGCGATAAGGACGCAG	Plasmid
MDA5-FUGW-R	CTAGCTAGCTCAGTCAGTTTCCAAGTTTTCTTCC	Plasmid
*mda5*-F	GGCATCATATTCACCCGGAC	Real-time PCR
*mda5*-R	ATTAGCAGATTGATCTGGCCGT	Real-time PCR
*lgp2*-F	TGGCAATGAATGCCTGCTAT	Real-time PCR
*lgp2*-R	ACCCCCTCCAGTGAGAATAC	Real-time PCR
S11-F	CCGTGGGTGACATCGTTACA	Real-time PCR
S11-R	TCAGGACATTGAACCTCACTGTCT	Real-time PCR
*svcv-g*-F	CGACCTGGATTAGACTTG	Real-time PCR
*svcv-g*-R	AATGTTCCGTTTCTCACT	Real-time PCR
EPC-*ifn-1*-F	ATGAAAACTCAAATGTGGACGTA	Real-time PCR
EPC-*ifn-1*-R	GATAGTTTCCACCCATTTCCTTAA	Real-time PCR
EPC-*mx*-F	GGCTGGAGCAGGTGTTGGTATC	Real-time PCR
EPC-*mx*-R	TCCACCAGGTCCGGCTTTGTTAA	Real-time PCR
EPC-*viperin*-F	AGCGAGGCTTACGACTTCTG	Real-time PCR
EPC-*viperin*-R	GCACCAACTCTCCCAGAAAA	Real-time PCR
EPC-*isg15*-F	ACAGTCGGTGAACTCAAGCAAGTC	Real-time PCR
EPC-*isg15*-R	CGTAACTGCTGAGGCTTCTGGAAT	Real-time PCR
EPC-EF-1α-F	AAGAGCGTTGAGAAGAAAG	Real-time PCR
EPC-EF-1α-R	GAGTGCCCAGGTTTAGAG	Real-time PCR

### Cell culture and transfection

Epithelioma papulosum cyprini (EPC) cell line is derived from fathead minnow (*Pimephales promelas*), which is widely used in fish disease research. 293T cell line is a derivative of human embryonic kidney 293 cells commonly used to detect interactions between proteins. EPC and 293T cells were generously donated by Yuguang Qin (Shandong Freshwater Fisheries Research Institute, China) and Guangxun Meng (Institute Pasteur of Shanghai, Chinese Academy of Sciences, China), respectively. EPC cells were cultured in Medium 199 (Gibco, Grand Island, NY, USA) supplemented with 10% fetal bovine serum (FBS, Gibco, Grand Island, NY, USA) and cultivated at 25°C without CO_2_. 293T cells were grown in Dulbecco’s modified Eagle’s medium (DMEM) (Gibco, Grand Island, NY, USA) supplemented with 10% FBS (Gibco, Grand Island, NY, USA), 100 U/mL penicillin and 100 μg/mL streptomycin (Life Technologies, Grand Island, NY, USA) and maintained at 37°C with 5% CO_2_. Target genes were transfected into 293T and EPC cells using Lipofectamine 2000 (Invitrogen, Carlsbad, CA, USA) and jetPRIME reagent (Polyplus, Illkirch, France), respectively.

### Gene cloning and plasmid construction

Plasmid enhanced green fluorescent protein (pEGFP)-N1 plasmid was purchased from Clontech (Mountain View, CA, USA). The plasmids of pmCherry-N1, pFUGW-2FLAG and pCMV-HA were generously donated by Guangxun Meng. To construct the pEGFP-*Cc*MDA5 and pEGFP-*Cc*LGP2 fusion vector, the whole open reading frame of *Cc*MDA5 (GenBank accession number: KM374815.1) and *Cc*LGP2 (GenBank accession number: KM374816.1) were produced using PCR and digested with the corresponding restriction enzymes (*Hind*III/*Bam*HI for *Cc*MDA5 and *Kpn*I/*Sma*I for *Cc*LGP2). The digested sequence was ligated into pEGFP-N1 using T_4_ DNA ligase (Life Technologies, Carlsbad, CA, USA). The other recombinant plasmids of *Cc*MDA5 and *Cc*LGP2 used in this study were cloned with the same method. The *ifn-1* promoter luciferase reporter plasmid was constructed as described previously (27).

### Confocal microscopy

EPC cells were seeded onto coverslips in a 24-well plate overnight before being transfected with EGFP-*Cc*MDA5 and mCherry-*Cc*LGP2 recombinant plasmids as depicted in the figures. The cells were rinsed with PBS and fixed with 4% PFA (paraformaldehyde) for 30 min at room temperature. Images were obtained using a laser scanning confocal microscope (Leica SP8, Wetzlar, Germany) after staining with 4’,6-diamidino-2-phenylindole (DAPI, Sigma Aldrich, St. Loius, MO, USA) for 10 min at room temperature.

### Luciferase activity assays

293T cells in a 96-well plate were co-transfected with a luciferase reporter plasmid (*ifn-1*-Luc), pRL-TK Renilla luciferase plasmid, target gene recombinant plasmid and the control vectors. After 48 h of transfection, cells were lysed using Dual-Glo^®^ Reagent (Promega, Madison, WI, USA). *Firefly* luminescence was measured after 10 min of incubation at room temperature. Dual-Glo^®^ Stop & Glo^®^ Reagent (Promega, Madison, WI, USA) were further combined in an equivalent volume. Ten minutes later, the activity of *Renilla* luciferase was determined. The *Firefly* luciferase/*Renilla* luciferase ratio was calculated following the manufacturer’s instructions.

### Crystal violet staining

EPC cells were cultivated in a 24-well plate and transfected with 0.5 μg of *Cc*MDA5/*Cc*LGP2 recombinant plasmid or the empty vector. After transfection for 24 h, the cells were infected with SVCV (10^6^ TCID_50_/mL, 30 μL) for twenty-four hours. Then the cells were washed with PBS, fixed with 4% PFA and dyed with 1% crystal violet for 30 min at room temperature.

### Coimmunoprecipitation and immunoblot analysis

Immunoprecipitation and immunoblotting were performed as described previously (28). Briefly, 293T cells seeded in 6-well plates were collected after 48-h transfection with the indicated plasmids. The cells were washed with PBS and lysed using lysis buffer (50 mM Tris-HCl, 15 mM ethylenediamine tetraacetic acid with pH 8.0, 150 mM NaCl, 1 mM NaF, 1 mM Na_3_VO_4_, and 1% Nonidet P-40) with protease inhibitor cocktail (Roche, Switzerland) at 4°C. After being centrifugated for 10 min at 12,000 × *g*, the supernatant was collected and transferred into a fresh tube. The remaining supernatants were immunoprecipitated using anti-FLAG conjugated agarose (Sigma-Aldrich, St. Louis, MO, USA) at 4°C overnight with constant agitation. The immunoprecipitated proteins were collected on the following day. After washing four times with lysis buffer, the complex was resuspended with 2 × sodium dodecyl sulfate (SDS) loading buffer (as the IP sample) and heated at 95°C. Subsequently, the whole-cell lysates and IP samples were separated by 10% SDS-polyacrylamide gel electrophoresis and transferred into a polyvinylidene fluoride membrane (Millipore, Bedford, MA, USA). The remaining steps of immunoblot were the same as previously stated (28).

### Statistical analysis

Statistical analysis was performed using GraphPad Prism 7.0 software. All data were presented as mean ± standard deviations (SD). Comparisons in experiments between two groups were assessed by t-test and a one-way analysis of variance was used in more than two groups. *P* < 0.05 was considered statistically significant.

## Results

### RNA Sequencing

A total of 18 samples (gills, foregut and hindgut tissues from the control and SVCV-infected group) were sequenced to screen for the critical genes involved in the immune response to SVCV infection. After Illumina sequencing, a total of 941,722,336 reads were identified. Furthermore, 932,378,600 clean reads were selected after quality control by filtering low-quality sequences. The benchmark for sequencing quality Q_30_ > 90%. Among the 18 samples, more than 80% of the clean reads were successfully mapped to the reference genome (**Table 2**). These results indicate that the transcriptome data were of high quality and the obtained unigenes were reliable and suitable for annotation analysis.

**Table 2:** Statistical table of sequencing results.

Sample	Raw reads	Clean reads	Q30 (%)	GC content (%)	Mapped ratio
C_fg_1	48084306	47577522	95.04	48.31	81.74%
C_fg_2	52064822	51527560	94.87	48.26	81.26%
C_fg_3	47616880	47196752	95.24	48.6	81.36%
C_gi_1	51652546	51122406	94.89	48.04	81.68%
C_gi_2	55902254	55416522	94.91	47.83	81.88%
C_gi_3	59634594	59070282	94.36	47.53	81.66%
C_hg_1	53586944	53078038	95.23	48.58	81.11%
C_hg_2	49098842	48628734	94.65	48.23	81.31%
C_hg_3	48022054	47581770	95.04	48.55	80.81%
SV_fg_1	55600984	55038750	94.71	48.85	81.56%
SV_fg_2	52273776	51711268	94.97	48.91	81.78%
SV_fg_3	55782616	55156310	94.88	48.73	81.45%
SV_gi_1	51492874	50939220	94.53	48.06	81.29%
SV_gi_2	50142832	49660294	95.14	49.33	80.27%
SV_gi_3	53225374	52661382	94.69	48.3	81.61%
SV_hg_1	51583236	51136824	95.35	48.19	81.29%
SV_hg_2	54249184	53749026	95.06	48.36	81.95%
SV_hg_3	51708218	51125940	94.41	48.67	81.16%
Total	941722336	932378600	>94%		≥80%

### Identification of DEGs

**Figure 1:**
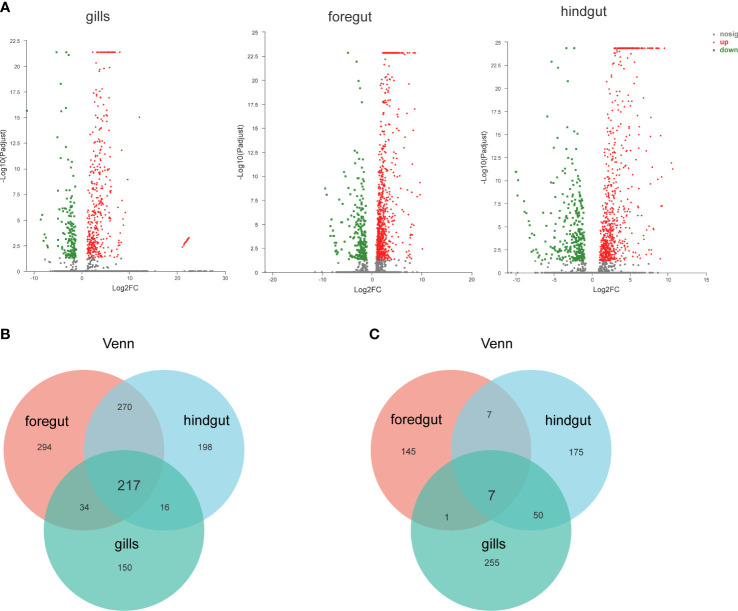
Identification and analysis of differentially expressed genes (DEGs). **(A)** Volcano plot of DEGs from the transcriptome in carp gills, foregut and hindgut between the control and spring viremia of carp virus (SVCV)-infected groups. **(B)** Venn diagrams representing the number of transcripts co-upregulated DEGs for each sample. **(C)** Venn diagrams representing the number of transcripts co-downregulated DEGs for each sample.

To study the genetic variation in response to SVCV, DEGs were screened by comparing transcriptome data. As presented in the volcanic plots (**Figure 1A**), a total of 2,645 DEGs were identified between the control and SVCV-infected groups. Among the 2,645 DEGs, 417 up-regulated and 160 down-regulated genes were detected in the gills, 815 up-regulated and 239 down-regulated genes were found in the foregut and 701 up-regulated and 313 down-regulated genes were identified in the hindgut, respectively. The DEGs induced by SVCV in the gills, foregut and hindgut are depicted in the Venn diagram. As presented in **Figures 1B, C**, 217 up-regulated and 7 down-regulated DEGs were common in the gills, foregut and hindgut.

### GO and KEGG enrichment analyses of upregulated DEGs

**Figure 2:**
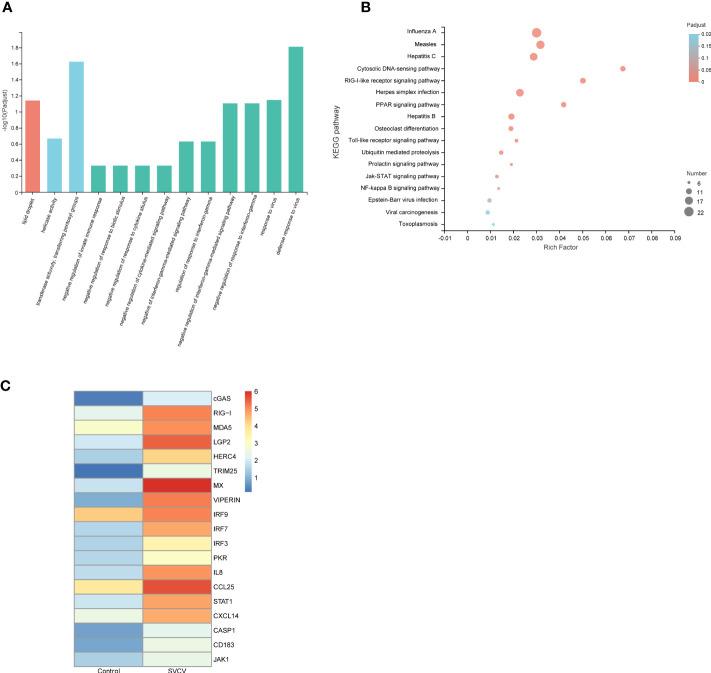
Function analysis of DEGs. **(A)** The gene ontology (GO) enrichment analysis of co-upregulated DEGs in gills, foregut and hindgut between the control and SVCV-infected groups. The X-axis indicates the GO terms, and the Y-axis indicates the degree of enrichment in this term. **(B)** The Kyoto Encyclopedia of Genes and Genomes (KEGG) enrichment analysis of co-upregulated DEGs in gills, foregut and hindgut. The X-axis indicates the rich factors of corresponding pathways, and the Y-axis indicates the pathway names. The different colors of the dots represent the q-value, and the number of DEGs in each pathway is represented by the size of the dots. **(C)** A heatmap plot of DEGs involved in infectious diseases and immune-related pathways that are enriched in the control group versus the SVCV-infected group.

GO and KEGG function enrichment analyses were used to better understand the biological roles of DEGs observed in the gills, foregut and hindgut. GO enrichment analysis revealed that the identified upregulated DEGs were significantly enriched in terms associated with innate immune response and host responses to the virus, such as defense response to the virus (biological process) and transferase activity, transferring pentosyl groups (molecular function) (**Figure 2A**). Furthermore, to determine the putative biochemical pathways of important upregulated DEGs, we identified the top 20 pathways using KEGG enrichment analysis. As depicted in **Figure 2B**, the upregulated DEGs were also significantly enriched in infectious diseases and pathways associated with innate immunity, such as Influenza A (22 genes), RIG-I-like signaling pathway (11 genes), Cytosolic DNA-sensing pathway (10 genes), PPAR (Peroxisome proliferator-activated receptors) signaling pathway (10 genes), Toll-like receptor signaling pathway (7 genes) and NF-κB signaling pathway (6 genes). The GO and KEGG function enrichment analyses were performed to analyze the respective identified upregulated DEGs in the gills, foregut and hindgut (**Supplementary Figure 1**). Furthermore, we analyzed the upregulated DEGs enriched in infectious diseases and pathways associated with innate immunity and constructed a heatmap. It was revealed that many RLR signaling pathway molecules, including LGP2, MDA5, IRF3 and IRF7, were significantly upregulated (**Figure 2C**). RLR signaling pathway has been reported to play pivotal roles in the antiviral innate immune response to RNA virus. Considering the controversial roles of LGP2 regulating MDA5 in various viral infections, we selected *Cc*MDA5 and *Cc*LGP2 for further analysis.

### The validation of DEGs

**Figure 3:**
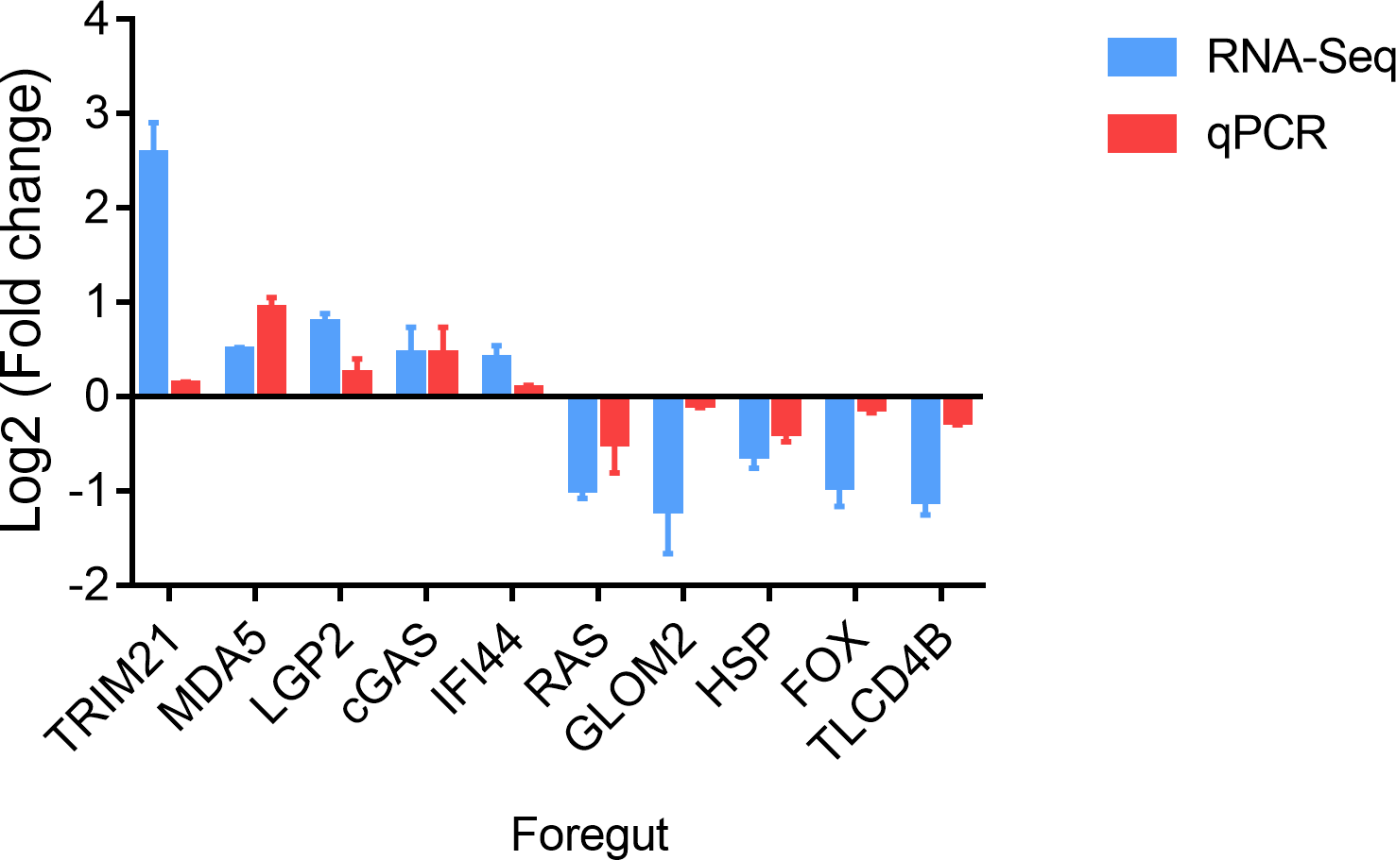
Validation of DEGs in the foregut. The mRNA levels of DEGs were normalized to the level of the S11 gene.

To verify the reliability of the transcriptome data, ten genes were randomly selected, and qPCR was performed in the foregut. These genes comprised tripartite motif-containing protein 21 (TRIM21), MDA5, LGP2, cyclic GMP-AMP synthase (cGAS), interferon-induced protein 44 (IFI44), ras-related GTP-binding protein (RAS), golgi membrane protein 2 (GLOM2), heat shock factor protein (HSP), forkhead-box (FOX) and TLC domain-containing protein 4-B (TLCD4B). As depicted in **Figure 3**, the expression of TRIM21, MDA5, cGAS, LGP2, and IFI44 was increased and the expression of RAS, GLOM2, HSP, FOX and TLCD4B was decreased in the foregut following SVCV infection. Consequently, the confirmed expression profile of DEGs was consistent with obtained RNA-seq analysis.

### *Cc*MDA5-mediated signaling inhibits SVCV infection

**Figure 4:**
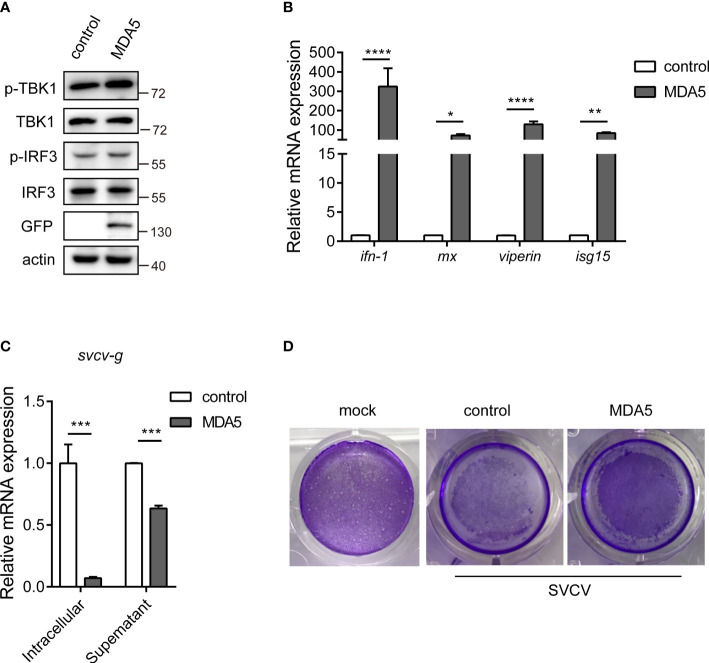
The effect of *Cc*MDA5 in the antiviral immune responses. **(A, B)** Empty vector or GFP-tagged *Cc*MDA5 plasmid was transfected into epithelioma papulosum cyprini (EPC) cells for 24 h. **(A)** Western blot was performed to test the phosphorylation of TBK1 and IRF3. β-Actin was used as an internal reference. **(B)** Total RNA was extracted to detect *interferon-1* (*ifn-1*), *myxovirus resistance* (*mx*), *viperin*, and *interferon-stimulated gene 15* (*isg15*) using qPCR. Data are represented as fold induction relative to the transcriptional level in control, which was set to 1. Mean ± SD (n = 3). **(C, D)** EPC cells were transfected with the above-mentioned plasmids. At 24 h post-transfection, the cells were stimulated with SVCV or Medium 199 and incubated at 25°C for 24 h. **(C)** Cell samples and supernatants were collected. The mRNA expression of the *svcv-g* gene was detected using qPCR. **(D)** The cell monolayers were stained with crystal violet, and CPEs were observed. The mock group represented the cells without treatment. Each experiment was conducted thrice. *P < 0.05, **P < 0.01, ***P < 0.001 and ****P < 0.0001.

To describe the potential role of *Cc*MDA5, we overexpressed *Cc*MDA5 in EPC cells and assessed the expression of the downstream antiviral molecules. As illustrated in **Figure 4A**, Western blot results revealed that *Cc*MDA5 overexpression induced phosphorylation of TBK1 (p-TBK1) and IRF3 (p-IRF3) in EPC cells. Additionally, overexpression of *Cc*MDA5 caused a marked upregulation of *interferon-1* (*ifn-1*), *myxovirus resistance* (*mx*), *viperin* and *interferon-stimulated gene 15* (*isg15*) (324.25-fold, 72.07-fold, 129.99-fold and 84.15-fold, respectively), compared with the control cells (**Figure 4B**). To identify the role of *Cc*MDA5 in virus proliferation, the mRNA expression of the *svcv-g* gene and virus-induced cytopathic effects (CPEs) were explored. Compared with the control cells, *Cc*MDA5 significantly decreased the transcriptional level of the *svcv-g* gene and CPEs (**Figures 4C, D** **)**. These results revealed that *Cc*MDA5 activates IFN signaling and inhibit virus proliferation.

### *Cc*LGP2 positively regulates *Cc*MDA5 inducing *ifn-1* expression

**Figure 5:**
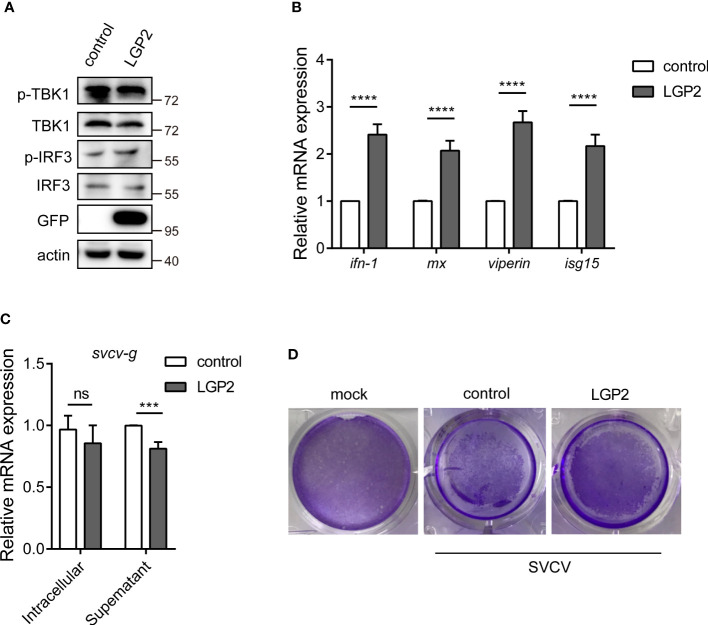
*Cc*LGP2 enhances the IFN activity and inhibits viral replication. Empty vector or GFP-tagged *Cc*LGP2 plasmid were transfected into EPC cells for 24 h. Further, the protein and RNA were extracted to detect downstream signaling molecules by Western blot **(A)** and qPCR **(B)**. **(C, D)** EPC cells were transfected with the above-mentioned plasmids. Twenty-four hours post-transfection, the cells were infected with SVCV and incubated at 25°C for 24 h. **(C)** Cell samples and supernatants were collected, and the expression of *svcv-g* was detected using qPCR. **(D)** The cell monolayers were stained with crystal violet, and CPEs were observed. Each experiment was conducted thrice. ***P < 0.001, ****P < 0.0001 and ns indicates no significant difference.

Previous studies have revealed that teleost LGP2 exhibited different antiviral activities against viral infection. To describe the potential role of LGP2 in common carp, we overexpressed *Cc*LGP2 in EPC cells. As illustrated in **Figures 5A, B**, the phosphorylation of TBK1 and IRF3 and the transcription of *ifn-1*, *mx*, *viperin* and *isg15* were significantly enhanced after overexpression of *Cc*LGP2. Additionally, *Cc*LGP2 also reduced the replication of the *svcv-g* gene and CPEs (**Figures 5C, D** **)**.

**Figure 6:**
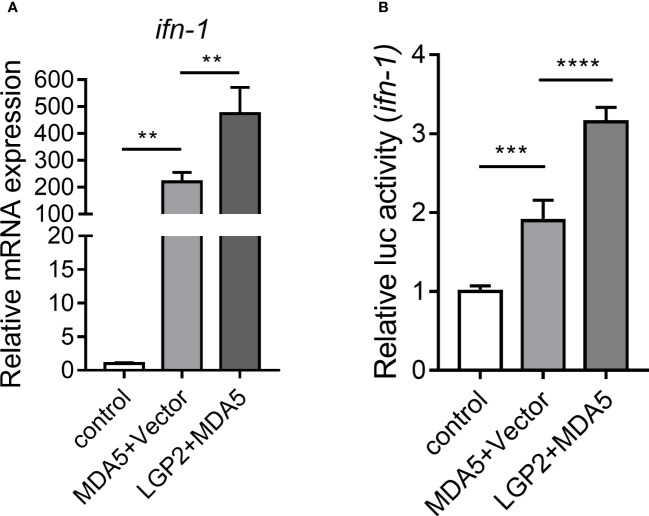
*Cc*LGP2 activates the *Cc*MDA5-inducing *ifn-1*. **(A)** Empty vector or FLAG-tagged *Cc*MDA5 plasmid was co-transfected with GFP-tagged *Cc*LGP2 plasmid into EPC cells for 24 h. Total RNA was extracted to detect *ifn-1*. Data are represented as fold induction relative to the transcriptional level in control, which was set to 1. **(B)** 293T cells were co-transfected with expression vectors for *Firefly* luciferase reporter gene with *ifn-1* promoter, *Renilla* luciferase and target gene for 48 h. The relative activity of *ifn-1* was the ratio of *Firefly* fluorescence to *Renilla* fluorescence. Mean ± SD (n = 3). Each experiment was conducted thrice. **P < 0.01, ***P < 0.001 and ****P < 0.0001.

To further explore the effect of *Cc*LGP2 on the *Cc*MDA5-mediated signaling pathway, qPCR and reporter assay were performed in EPC and 293T cells, respectively. As illustrated in **Figure 6A**, in comparison with the *Cc*MDA5 group, the expression of *ifn-1* was significantly up-regulated following co-transfection of *Cc*LGP2 and *Cc*MDA5. Additionally, *Cc*LGP2 significantly enhanced *Cc*MDA5-induced *ifn-1* promoter activation in 293T cells (**Figure 6B**). These results revealed that *Cc*LGP2 acts as the positive regulator in the MDA5-mediated *ifn-1* activation.

### Association between *Cc*LGP2 and *Cc*MDA5

**Figure 7:**
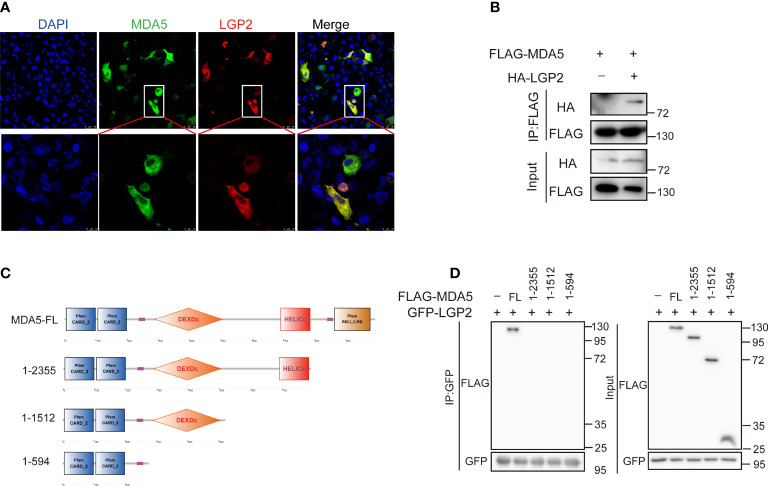
*Cc*LGP2 protein interacts with *Cc*MDA5 through the RD domain. **(A)** EPC cells were transfected for 24 h with plasmids expressing GFP-tagged *Cc*MDA5 and mCherry-tagged *Cc*LGP2. After staining with DAPI, the coverslip was observed under a laser scanning confocal microscope. **(B)** As indicated in the figure, FLAG-tagged *Cc*MDA5 and HA-tagged *Cc*LGP2 were separately transfected into 293T cells. The cell lysates were immunoprecipitated with anti-FLAG antibodies and further immunoblotted with anti-FLAG and anti-HA antibodies. The control group was transfected with FLAG-tagged *Cc*MDA5 and empty vector pCMV-HA. **(C)** Schematic diagram of full-length and mutant CcMDA5. **(D)** GFP-tagged *Cc*LGP2 and different truncations of *Cc*MDA5 were transfected into 293T cells for 48 h as indicated in the figure. The cell lysates were immunoprecipitated with anti-GFP antibodies and further immunoblotted with anti-FLAG and anti-GFP antibodies. All the experiments were repeated at least thrice.

To clarify the regulatory mechanism between *Cc*LGP2 and *Cc*MDA5, confocal microscopy and coimmunoprecipitation experiments were conducted. It was observed that *Cc*LGP2 colocalized with *Cc*MDA5 in the EPC cells cytoplasm (**Figure 7A** and **Supplementary Figure 2**). The CO-IP assay further verified their association, indicating an interaction between *Cc*LGP2 and *Cc*MDA5 (**Figure 7B**). Additionally, we investigated the domain of *Cc*MDA5 that interacted with *Cc*LGP2. As depicted in **Figures 7C, D**, different truncations of *Cc*MDA5 were generated, and the CO-IP assay revealed that *Cc*LGP2 was associated with the full-length of *Cc*MDA5, albeit not with other truncations, suggesting that *Cc*LGP2 bound with the C-terminal RD of *Cc*MDA5. Overall, these observations indicate that *Cc*LGP2 interacts with *Cc*MDA5 *via* the RD.

## Discussion

Teleost mucosal tissues serve as the first line of defense in innate immunity and play an essential role against pathogen infection (29, 30). Previous studies have demonstrated that the microbial composition of mucosal tissues is changed, and the mucosal immune response is also activated during pathogen stimulation (31). Although studies have illustrated the changes in microbial composition following pathogen infection and environment changes (31, 32), the antiviral mechanism of mucosal tissues remains elusive. Therefore, in this study, we selected carp mucosal immune tissues (gills, foregut and hindgut) for RNA-Seq analysis to explore the changes in the host immune system following SVCV infection. Following Illumina sequencing, 932,378,600 high-quality reads and 2,645 DEGs were identified.

KEGG analysis revealed that most of the co-upregulated DEGs were involved in pathogenic infection-related pathways, such as Influenza A, Cytosolic DNA-sensing pathway, RIG-I-like signaling pathway, PPAR signaling pathway and Toll-like receptor signaling pathway. As reported in previous studies, these pathways play essential roles in antiviral immune response (33–35). In transcriptome profiling, there were several genes identified as significantly up-regulated genes, including RIG-I, MDA5, LGP2, cGAS, IRF3 and IRF7. cGAS is the major sensor for detecting DNA and RNA viruses (36). Mice with cGAS deficiency have a higher mortality rate and viral titer (37). In zebrafish, cGAS knockdown inhibits *Edwardsiella tarda*-induced production of immunoglobulin (Ig)Z in gills and skin (38). Therefore, cGAS plays a crucial part in mucosal immunity against SVCV in common carp. IRFs are a family of transcription factors, playing important and diverse roles in viral infections by activating IFNs (39). IRF3 and IRF7 are the most important regulators of type I IFN against viral infection (40, 41). Hence, the initiation of transcription of IRF3 and IRF7 regulates IFN signaling against SVCV infection. RIG-I, MDA5 and LGP2, the members of RIG-I-like receptors, have been reported to be involved in the mucosal immune response against viral invasion (42). Additionally, our previous study has indicated a potential role of RIG-I in SVCV infection (26). Further studies are required to unravel the biological roles of MDA5 and LGP2 in SVCV infection.

Previous studies have demonstrated that MDA5, together with RIG-I, are cytoplasmic sensors of double-stranded RNAs (43, 44). MDA5 recruits the adaptor MAVS after interacting with viral RNA to initiate the production of IFN and antiviral molecules (45). The role of fish MDA5 in the induction of IFN has been well-elucidated during the last few decades (43, 46, 47). For instance, zebrafish MDA5 forms a complex with MAVS and then induces the IFN-I promoter activity (48). MDA5 from orange-spotted grouper activates the IFN promoter activity and interferon-stimulated response element (ISRE) activity and increases the transcription of IRF3, IRF7 and tumor necrosis factor receptor-associated factor 6 (TRAF6) (49). Consistent with these results, *Cc*MDA5 overexpression not only significantly induced the phosphorylation of TBK1 and IRF3 but also promoted the expression of *ifn-1* and ISGs. IFNs are a group of vital cytokines, which can induce a wide range of ISGs to limit virus replication and spread (50) (51). For instance, gibel carp IFNα remarkably inhibits cyprinid herpesvirus 2 (CyHV-2) propagation by activating signal transducer and activator of transcription 1 (STAT1), MX and protein kinase R (PKR) (52). Similarly, in EPC cells, *Cc*MDA5 can reduce the expression level of the *svcv-g* gene and inhibit SVCV replication by inducing the expression of *ifn-1*, *mx*, *viperin* and *isg15*. MDA5-mediated antiviral signaling pathways are conserved in mammals and teleost.

LGP2, the third member of the RLR family, possesses the DExD/H box RNA helicase domain that is important for inducing IFN-β response (11). However, LGP2 lacks the CARD domain that is required for signaling, which is responsible for the controversial roles of LGP2 in RLR-mediated signaling (53). To date, numerous pieces of evidence have illustrated the positive regulatory mechanism between the LGP2 and MDA5 (54). For instance, mammalian LGP2 can amplify the MDA5-mediated signaling pathway by augmenting the ability of MDA5 to form stable filaments (54, 55). In teleost, LGP2 plays a positive role in viral infection in Japanese flounder (53) and miiuy croaker (56). In line with these results, *Cc*LGP2 overexpression significantly upregulated TBK1, IRF3, *ifn-1*, *mx*, *viprein* and *isg15*, and promoted MDA5-mediated *ifn-1* activation. In contrast, LGP2, in mandarin fish (57), grouper (58) and rainbow trout (59) functions as a negative regulator in antiviral response by decreasing the transcription of IRF3, NF-κB and IFN-β. Recent studies have revealed that LGP2 is a bifunctional protein and yields a biphasic effect on MDA5 signaling (60, 61). Hence, the role of fish LGP2 in antiviral response may be associated with fish and virus species.

Conclusively, the transcriptome profiling in the common carp gills, foregut and hindgut after SVCV infection was elaborated. Two key genes, *Cc*MDA5 and *Cc*LGP2, were identified post SVCV infection. *Cc*LGP2 acted as a positive regulator in MDA5-induced *ifn-1* activation. Collectively, the study will provide a better understanding for further investigation into the innate immune mechanism in common carp fight against viral infection.

## Data availability statement

The data presented in the study are deposited in National Center for Biotechnology Information (NCBI) Short Read Archive (SRA) under accession number PRJNA873930. (https://www.ncbi.nlm.nih.gov/bioproject/PRJNA873930).

## Ethics statement

The animal study was reviewed and approved by Animal Experimental Ethics Committee of Shandong Normal University.

## Author contributions

SS and GY conceived and designed the experiments. RL, YN and performed the experiments and analyzed the data. YQ and HL helped with the experiments. SS and RL wrote the manuscript. All authors contributed to the article and approved the submitted version.

## Conflict of interest

The authors declare that the research was conducted in the absence of any commercial or financial relationships that could be construed as a potential conflict of interest.

## Publisher’s note

All claims expressed in this article are solely those of the authors and do not necessarily represent those of their affiliated organizations, or those of the publisher, the editors and the reviewers. Any product that may be evaluated in this article, or claim that may be made by its manufacturer, is not guaranteed or endorsed by the publisher.
