# Supplementary figures and images for: Transcriptome analysis identifies LGP2 as an MDA5-mediated signaling activator following spring viremia of carp virus infection in common carp (Cyprinus carpio L.)
Source: Front Immunol. 2022 Oct 18;13:1019872. doi: 10.3389/fimmu.2022.1019872 (PMC9623169; doi:10.3389/fimmu.2022.1019872)

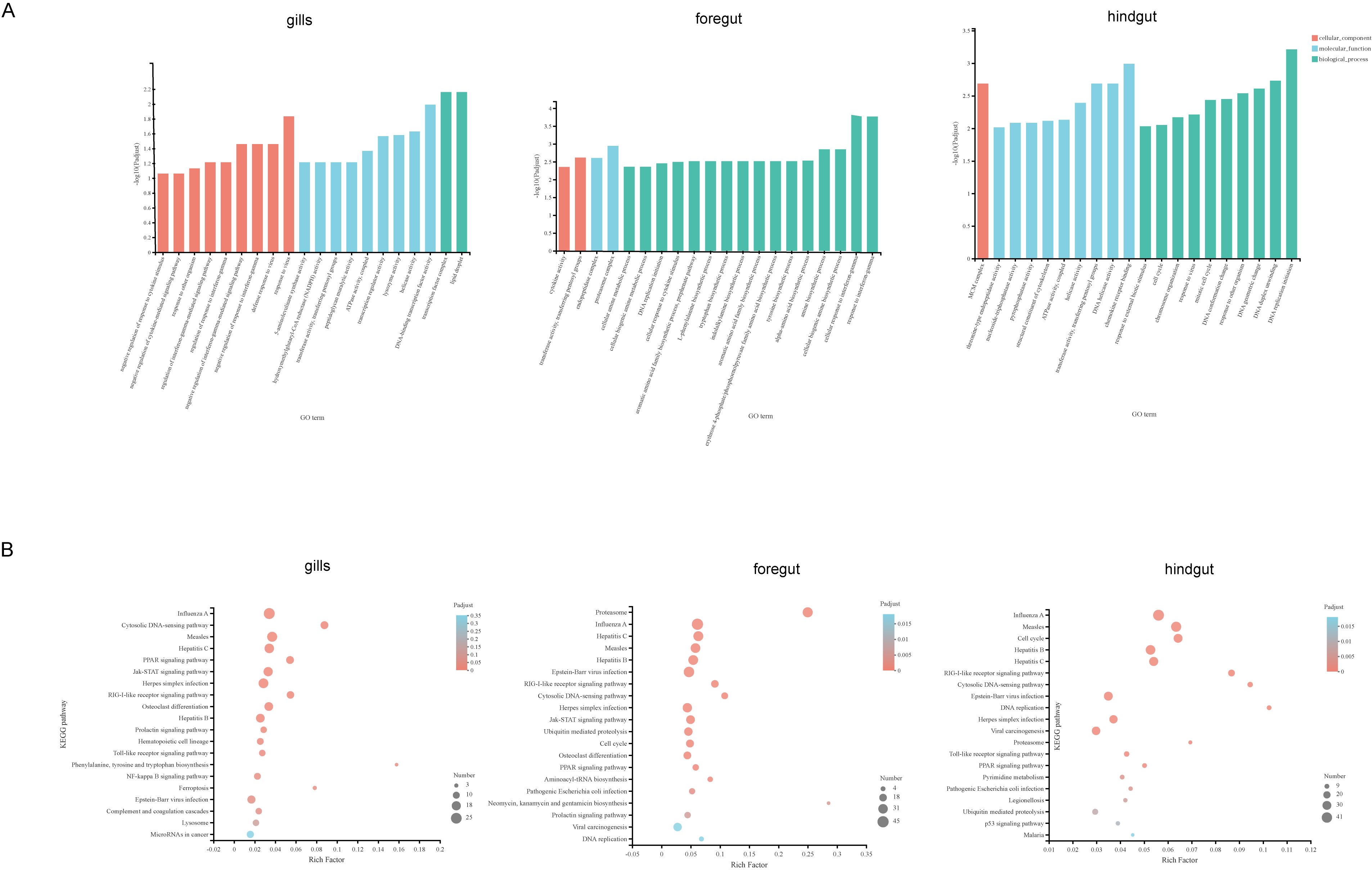

Supplement: Supplementary file 1 [file Image_1.tif]

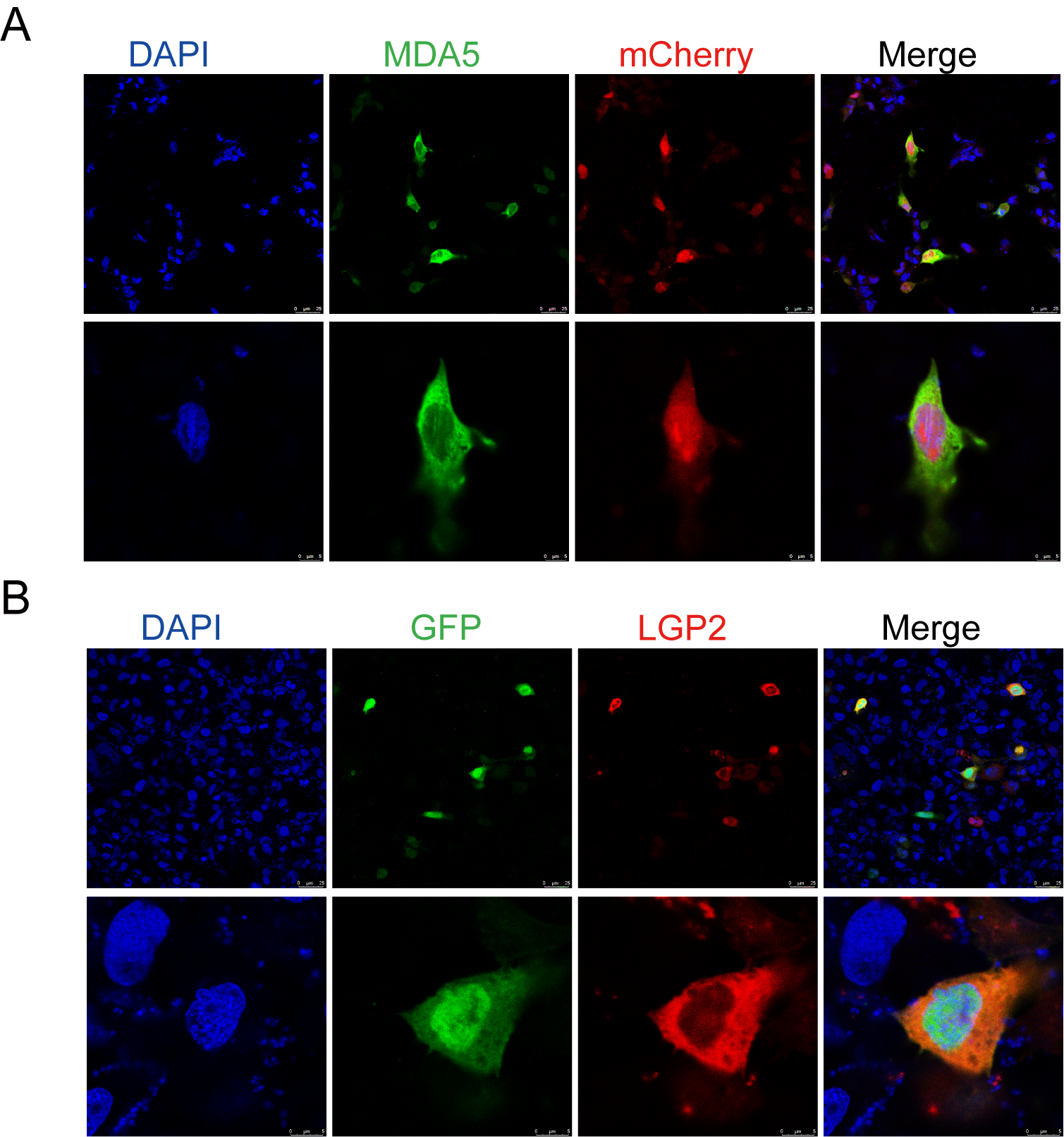

Supplement: Supplementary file 2 [file Image_2.tif]
